# Recent progress in melting heat phenomenon for bioconvection transport of nanofluid through a lubricated surface with swimming microorganisms

**DOI:** 10.1038/s41598-022-12230-4

**Published:** 2022-05-19

**Authors:** Marei Saeed Alqarni, Sumeira Yasmin, Hassan Waqas, Shan Ali Khan

**Affiliations:** 1 Department of Mathematics, College of Sciences, King Khalid University, Abha, 61413 Saudi Arabia; 2 Department of Mathematics, Government College University Faisalabad, Faisalabad, 38000 Pakistan

**Keywords:** Biophysics, Engineering, Materials science, Mathematics and computing, Nanoscience and technology

## Abstract

The cooling of numerous microelectronic devices has become a need in today's world. Nanofluids, a novel type of heat transport fluid containing nano-sized particles embedded in a host liquid, were developed a few years ago. Impact of ultra-fine nanoparticles with oil, water, or ethylene glycol produces these fluids. Nano-liquids have a variety of applications, including engine cooling, electronic devices, biomedicine, and the manufacture of thermal exchangers. The main objective of current research article is to scrutinizes theoretically, the effects of axisymmetric magnetohydrodynamic flow of bio-convective nanoliquid through a moving surface in the occurrence of swimming microorganisms. The idea of the envisaged model is improved by considering the consequence of thermal radiation, activation energy with generalized slip effects under convective boundaries. The present analysis is developed in the form of mathematical formulation and then solved numerically. The governing flow equations are transmuted into dimensionless nonlinear ODEs system by compatible similarity transformations and then integrated this so-formulated highly nonlinear problem numerically via bvp4c built-in scheme in MATLAB. The significance of influential parameters versus velocity field, temperature profile, concentration field and motile density of microorganism’s profile are examined with the aid of graphs and tabular data. The physical interpretation of outcomes highlight that the velocity receives increment for amplified mixed convection parameter. The thermal profile is found to be reducing with a greater Prandtl number. The concentration profile of nanoparticle boosts up for greater activation energy parameter. The microorganism’s profile is reduced via bioconvection Lewis number. This investigation contains the significance of bioconvection phenomenon, thermal radiation, slip effects and activation energy under convective boundary conditions. These impacts are used in axisymmetric, stagnation point flow of bioconvective magnetized nanofluid containing swimming gyrotactic motile microorganisms over a lubricated surface. The present analysis is not yet published.

## Introduction

Melting process^1^ has received a lot of attention because of its wide applications in techniques and manufacturing companies. Recent investigators and scientists have been inspired to create modern energy techniques and energy resources that use solar energy. They have given their undivided attention to the development of maintainable and lower-cost energy stored technology. This form of technology is associated to waste heat recovering, solar energy, as well as power/energy plants. There are three primary methods for storing energy. These would be chemical heat energy storing, latent heat power storing and rational heat energy storing. The greatest suitable method of energy processing is latent heat, which is obtained by varying the step of materials. Thermal power can be processed in a material through latent energy by melting and recovered by freezing. These implementations include molten solidification, microprocessor material development, sewage therapy welding, as well as casting of industrial processes, soil freezing, silicon water process and several others^2^. Epstein and Cho^3^ were firstly to investigate the melting phenomena on a smooth surface. Tamuli et al.^4^ scrutinized the melting performance of phase change material in an enclosure. Samantaray and Sarangi^5^ evaluated the melting phenomena of metallic silver nanocrystals (*AgN*) with $N$ = 108 to 4000 atoms utilizing molecular dynamics computations with configured engrained atomic technique potential parameters. Waqas et al.^6^ reported the cross-based nanofluid flow with non-linear thermal radiation, activation energy and melting phenomena. Waqas et al.^7^ expressed the enhancement in heat transport for bioconvection flow of second grade nanofluid with melting processes via a stretchable surface in the presence of motile microorganisms. The melting phenomenon with nanofluid is more important to improve the heat transfer.

Nanofluids^8^ are produced by the suspension of nanometer-sized particles (below 100 nm) in regular fluids including water. The practical examples of nanofluids include cooling systems, emulsification, ethanol glycol or tri-ethylene fluid, many lubricating oils, polymer formulations and certain popular bio-fluids. It has a wide range of applications involving microelectronic devices^9^, hybrid powered motors, diesel cells refrigerator, air conditioner vehicle, cooling device^10^, chiller, crushing system pharmacy method, boiler exhaust gases recovered, ultrasound field, high-power laser, nanoengineering field, micro-reactors^11^, welding cooled, cooling nuclear device^12^, electromagnetic tubes, space, thermal processing and drag reducing. Nanofluids can be produced at the manufacturing level using two major processes like one-step and two-step^13^. In one step process, the particle factories and absorbs in fluids medium at the same time, without proceeding via the mechanism of dehydration, storage, transport and dispersion of nanomaterials. As a result, the range of nanoparticles is decreased, and fluid stabilization is increased. In these specific instance of preparation, large-scale nanofluid necessitates advanced techniques are costly. On the other side, the two processes are commonly utilized in the manufacturing prepping of nanoparticles. According to this procedure, nanometer-size particles, fabrics, tubes and certain nanomaterials are developed in the presence of free powders. The definition of nanofluids was firstly given by Choi and Eastman^14^. He established this concept of nanoparticles in a fluid process. He has observationally shown that the thermal properties of regular fluid have improved through addition of nanoparticles. Buongiorno^15^ has introduced a nanofluid model to see thermo physical aspects of base fluids. Shamshuddin and Eid^16^ discussed the incompressible, steady, mixed convection flow of nanofluid with influence of Joule heating. Swain and Mahanthesh^17^ analyzed two-dimensional radiative magneto-nanofluid under Joule heating impact. Hayat et al.^18^ explored the Brownian motion coefficient and thermophoresis effect in flow of micropolar nanofluids under the thermal radiation impact. Mahanthesh and Mackolil^19^ addressed the implications of quadratic heat radiation effect and the quadratic Boussinesq assumption on the heat transport of a 36 nm Al_2_O_3_–H_2_O nanoparticles through a vertical surface. Eid and Mabood^20^ discussed the hydrothermal fluctuations of viscoelastic nanoliquid in a porous media across a moving surface. Many researchers have explored the properties of nanofluids against different geometries in studies^21–27^.

Activation energy is the minimum quantity of heat that is reasonable to enhance molecules or atoms to an environment in which chemical reactions or physiological transport can eventuate. Commonly, the connection among mass transport and chemical processes is very complicated and can also be examined at various fluid flowing and mass transport concentrations combined through the manufacturing and processing of reagent organisms. The several significant benchmarks are the organism that does not normally correspond to chemical processes through Arrhenius activation energy. In 1889, mathematicians Svante Arrhenius initially mentioned the term of activation energy. The minimum amount of energy conquers through atoms, molecules to active the chemical reaction^28^. The application of Arrhenius activation energy increased for chemical technology, geothermal, oil and water pigment mechanics. Naturally a convective binary chemical reaction is investigated by Bestman^29^. The Arrhenius model as modified by Tencer et al.^30^ as obeys:

$$k_{r} = B\left( {\frac{T}{{T_{\infty } }}} \right)^{n} \exp \left( { - \frac{{E_{a} }}{{k_{1} T}}} \right),$$

In above equation, $k_{r}^{2}$ is the comical reaction rate, $B$ the pre-exponential feature and $E_{a}$ the activation energy, $k_{1} = 8.61 * \frac{{10^{ - 5} ev}}{K}$ the Stefan Boltzmann constant and $n$ the fitted rate constant that arises in the distance connecting −1 to 1. The Arrhenius activation energy applications include medications or energy powers and oil as well as water emulsifiers. Khan and Alzahrani^31^ scrutinized the Jeffrey nanofluid flow with viscous dissipation against a curve surface involving entropy generation (EG) and activation energy. Awais et al.^32^ assessed the non-linear Boussinesq assumption in transport of hyperbolic tangential nanofluid across a moving surface with generation of entropy. Gotoh et al.^33^ described the Arrhenius activation energy of hydrogen extraction through carrier-selective interactions involving silicon oxide interlayer in high-efficiency of titanium oxide.

Microorganisms are more constructive in nanofluid to enhance heat transfer rate. The collision of nanoparticles is more important factor in the heat transfer rate. Microorganisms have performed an important role in enhancing human’s life specifically because of applications on the biomedical field^34^. The life is more difficult to lead without important microorganisms. Such organisms are very tiny to be seen even by a strong microscope, but they are too large for the atmosphere. Biofuels^35^, manufacturing and agricultural technologies, enzyme biomaterials, mass transport bioengineering and biomedical sciences are part of their involvement in life. Researchers are really involved in research about microorganisms. The macroscopic liquid convective movement induced through the density gradient is identified as "bioconvection" and is induced by the combined swimming of motile gyrotactic microorganisms. Such self-propelled gyrotactic microorganisms improved the basic fluid density through swimming in a specific direction resulting in bioconvection. The word "bioconvection" has been firstly used by Platt^36^ since the starting of flow behaviors found in dense communities of free-swimming micro-organisms (for example Tetrahymena, ciliates as well as flagellates). Kuznetsov^37^ investigated both non-oscillatory as well as oscillations nanofluids biothermal heat transfer in a horizontal limiting depth surface and examined the dependency of the Rayleigh number for the Rayleigh number nanomaterials as well as the Rayleigh number bioconvection. In the existence of motile microorganisms, Khan and Makinde^38^ explored the MHD transport of nanoparticles through heat and mass transfer around a vertical stretched surface. Xu and Pop^39^ achieved a further biologically practical result utilizing a passively supported nanofluid structure by studying the bioconvection movement of nanoparticles inside a horizontal channel. Chu et al.^40^ investigated Joule heating and Arrhenius activation energy in tangent hyperbolic bioconvective nanofluid flow. Alshomrani et al.^41^ addressed the impact of numerous slips on the swimming bioconvection flow of cross nanofluid through a wedge containing microorganisms. Wakif et al.^42^ observed modified Fourier's and Fick's laws for approximating mixed bioconvection flows of radiative-reactive Walters-B nanofluids carrying tiny nanoparticles with Lorentz force. Islam et al.^43^ scrutinized magnetohydrodynamic Darcy-Forchheimer nanofluid flow by stretching surface with convective conditions and gyrotactic motile microorganisms. Muhammad et al.^44^ considered the time-dependent flow of a rheological Carreau-type nanofluid with motile microorganisms through a wedge including velocity slip and thermal radiation aspects. Sheikholeslami^45^ discussed the computational analysis for exergy and entropy in nanofluid through porous medium. The phenomenon of bioconvection is examined on different fluids by numerous researchers can be seen in references^46–48^.

Based on the motivation and authors knowledge, this article has the following innovations:

1. In this paper, numerical solution of nanofluid flow is developed by applying bvp4c tool of MATLAB with shooting scheme.
2. Flow is generated through a lubricated surface.
3. Bioconvection impact with thermal radiation is investigated.
4. This scrutinization of bioconvective nanofluid has applications in bioengineering and biofuels, cancer therapy, and in mechanical problems.
5. In our knowledge, this may the first attempt to scrutinize the bioconvection aspects on magnetized nanofluid flow through lubricated surface under melting phenomenon and gyrotactic motile microorganisms.

## Mathematical description

### Flow of considered model and coordinates system

**Figure 1:**
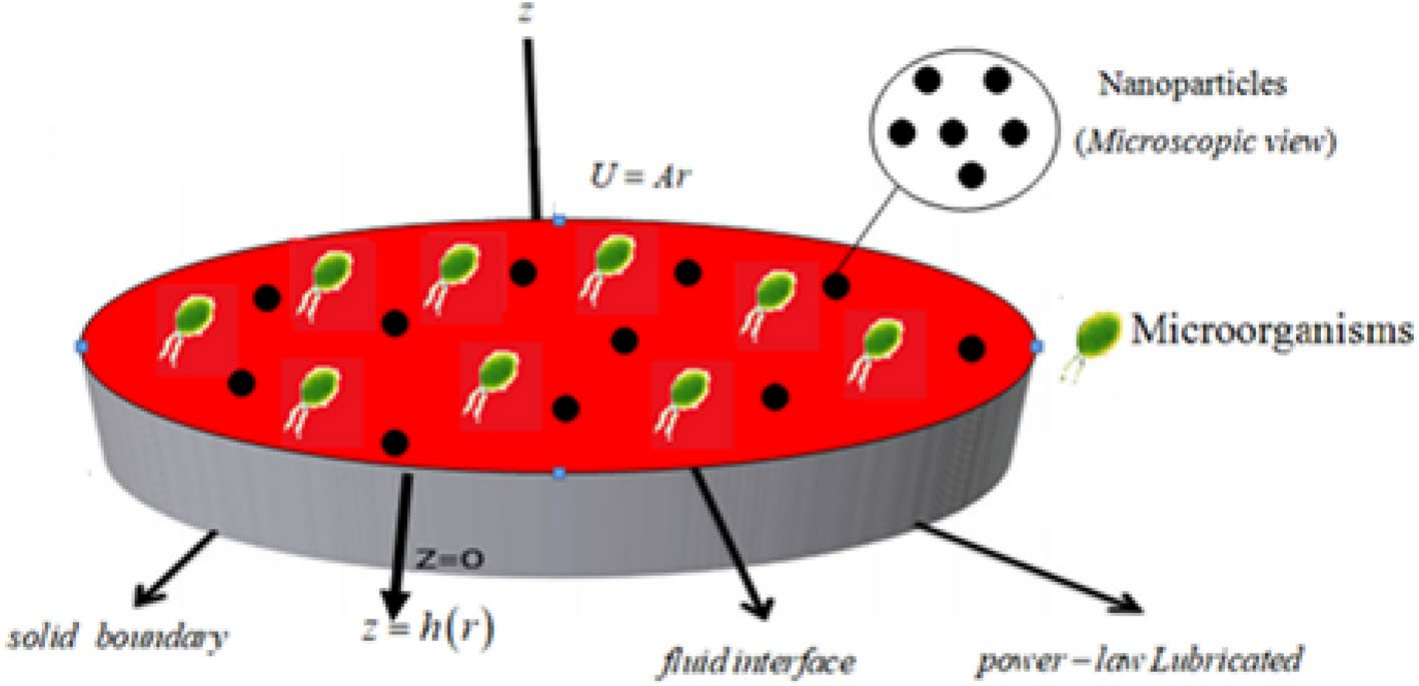
Schematic flow and coordinates system.

Here we developed a numerical solution to scrutinize the impact of melting phenomenon and thermal radiation on bioconvective axisymmetric flow of magnetized nanofluid containing swimming motile microorganisms past a lubricated surface under slip condition. Buongiorno’s model for nanofluid is utilized to aid the thermophoresis diffusion and Brownian motion impacts in energy as well as in concentration expressions. The flow is induced under convective and Neild’s boundary conditions. The components of velocity $\left[ {u1\left( {r,z} \right),0,u3\left( {r,z} \right)} \right]$ and the lubrication components $\left[ {U1\left( {r,z} \right),0,U3\left( {r,z} \right)} \right]$ are also considered. The fluid flow schematic diagram of current model is captured in Fig. 1. The surface is showered under the lubricated power-law generating a thin layer through varying thickness $h\left( r \right)$. The rate of flow $Q$ is expressed as:

1 $$Q = \int\limits_{0}^{h\left( r \right)} {2\pi rU1dz}$$

### Governing equations

The boundary layer governing flow equations are as follows:

2 $$\frac{\partial u1}{{\partial r}} + \frac{u1}{r} + \frac{\partial u3}{{\partial z}} = 0,$$

3 $$\begin{aligned} u1\frac{\partial u1}{{\partial r}} + u3\frac{\partial u1}{{\partial z}} = & - \frac{1}{\rho }\frac{\partial p}{{\partial r}} + \nu \left\{ {\frac{{\partial^{2} u1}}{{\partial r^{2} }} + \frac{\partial }{\partial r}\left( \frac{u1}{r} \right) + \frac{{\partial^{2} u1}}{{\partial z^{2} }}} \right\} - \frac{{\sigma^{**} \beta_{0}^{2} u1}}{\rho } \\ & \; + \frac{1}{{\rho_{f} }}\left[ {\begin{array}{*{20}l} {\left( {1 - C_{f} } \right)\rho_{f} \beta^{**} g^{*} \left( {T - T_{\infty } } \right) - \left( {\rho_{p} - \rho_{f} } \right)g^{*} \left( {C - C_{\infty } } \right)} \hfill \\ { - \left( {N - N_{\infty } } \right)g^{*} \gamma \left( {\rho_{m} - \rho_{f} } \right)} \hfill \\ \end{array} } \right], \\ \end{aligned}$$

4 $$u1\frac{\partial u3}{{\partial r}} + u3\frac{\partial u3}{{\partial z}} = - \frac{1}{\rho }\frac{\partial p}{{\partial z}} + \nu \left\{ {\frac{{\partial^{2} u3}}{{\partial r^{2} }} + \frac{{\partial^{2} u3}}{{\partial z^{2} }} + \left( \frac{1}{r} \right)\frac{\partial u3}{{\partial r}}} \right\},$$

5 $$u1\frac{\partial T}{{\partial r}} + u3\frac{\partial T}{{\partial z}} = \alpha \left( {\frac{1}{r}\frac{\partial T}{{\partial z}} + \frac{{\partial^{2} T}}{{\partial z^{2} }}} \right) + \tau \left( {D_{B} \frac{\partial T}{{\partial z}}\frac{\partial C}{{\partial z}} + \frac{{D_{T} }}{{T_{\infty } }}\left( {\frac{\partial T}{{\partial z}}} \right)^{2} } \right) - \frac{1}{{\rho C_{p} }}\frac{{\partial q_{r} }}{\partial z},$$

6 $$u1\frac{\partial C}{{\partial r}} + u3\frac{\partial C}{{\partial z}} = D_{B} \left( {\frac{1}{r}\frac{\partial C}{{\partial z}} + \frac{{\partial^{2} C}}{{\partial z^{2} }}} \right) + \frac{{D_{T} }}{{T_{\infty } }}\left( {\frac{1}{r}\frac{\partial T}{{\partial z}} + \frac{{\partial^{2} T}}{{\partial z^{2} }}} \right) - kr^{2} \left( {C - C_{m} } \right)\left( {\frac{T}{{T_{\infty } }}} \right)^{m} \exp \left( {\frac{{ - E_{a} }}{{K_{1} T}}} \right),$$

7 $$u1\frac{\partial N}{{\partial r}} + u3\frac{\partial N}{{\partial z}} + \left[ {\frac{\partial }{\partial z}\left( {N\frac{\partial C}{{\partial z}}} \right)} \right]\frac{{bW_{c} }}{{\left( {C_{\infty } - C_{m} } \right)}} = D_{m} {\frac{\partial }{\partial z}\left( {\frac{\partial N}{{\partial z}}} \right)},$$

Thermal radiative heat flux is denoted as

8 $$q_{r} = - \frac{{4\sigma^{*} }}{{3k^{*} }}\frac{{\partial T^{4} }}{\partial z},$$

here $k^{*}$ represents the mean absorption coefficient and $\sigma^{*}$ the Stefan Boltzmann constant. Therefore,

9 $$T^{4} = 4TT_{\nu }^{3} - 3T_{\infty }^{4} .$$

We get

10 $$\frac{{\partial q_{r} }}{\partial z} = - \frac{{16T_{\nu }^{3} \sigma^{*} }}{{3k^{*} }}\frac{{\partial^{2} T}}{{\partial z^{2} }}.$$

With no-slip conditions

11 $$\begin{aligned} U1 = & 0 = U3,\;\; - k\left. {\left( {\frac{\partial T}{{\partial z}}} \right)} \right|_{z = 0} = \rho \left[ {L + \left( {T_{m} - T_{0} } \right)c_{s} } \right]u3,\;\; - k\frac{\partial T}{{\partial z}} = h_{f} \left( {T_{m} - T} \right), \\ & \;\;D_{B} \frac{\partial C}{{\partial z}} + \frac{{D_{T} }}{{T_{\infty } }}\frac{\partial T}{{\partial z}}=0,\;\;N = N_{m} ,\;\;{\text{at}}\;\;z = 0, \\ \end{aligned}$$

with

12 $$U1 = 0\;\;\;{\text{for}}\;\;z \in \left[ {0,h\left( r \right)} \right].$$

The conditions of interfacial as^49^

13 $$u\frac{\partial u}{{\partial z}} = k\left( {\frac{\partial U}{{\partial z}}} \right)^{n} \left[ {1 - \beta^{*} k\left( {\frac{\partial U}{{\partial z}}} \right)^{n} } \right]^{{ - \frac{1}{2}}} .$$

Here $k$ is consistency index, $n$ power-law index and $\beta^{*}$ reciprocal of critical shear rate. Thus,

14 $$U1\left( {r,z} \right) = \frac{{\overset{\lower0.5em\hbox{$\smash{\scriptscriptstyle\frown}$}}{U} 1\left( r \right)sz}}{h\left( r \right)}.$$

For both fluids the velocity component $$\overset{\lower0.5em\hbox{$\smash{\scriptscriptstyle\frown}$}}{U} 1\left( r \right)$$ is at the interfacial situation. The thickness is articulated as

15 $$h\left( r \right) = \frac{Q}{{\pi r\overset{\lower0.5em\hbox{$\smash{\scriptscriptstyle\frown}$}}{U} 1\left( r \right)}}$$

Putting the values in Eqs. (12) and (13) and $$\overset{\lower0.5em\hbox{$\smash{\scriptscriptstyle\frown}$}}{U} 1 \cong u$$ in (11), we obtain

16 $$\frac{\partial u}{{\partial z}} = \frac{k}{\mu }\left( {\frac{\pi }{Q}} \right)^{n} r^{n} u^{2n} \left[ {1 - \beta^{*} k\left( {\frac{\pi }{Q}} \right)^{n} r^{n} u^{2n} } \right]^{{ - \frac{1}{2}}} .$$

The continuity for velocities of fluid and lubricated on the interfacial constraints specified as

17 $$u3\left( {r,h\left( r \right)} \right) = U3\left( {r,h\left( r \right)} \right).$$

From Eq. (12), we have

18 $$u3\left( {r,h\left( r \right)} \right) = 0.$$

By the interfacial constraints, the pressure distributed of pressure measured as

19 $$p\left( {r,h\left( r \right)} \right) = - \rho \frac{{A^{2} r^{2} }}{2},$$

with

20 $$u1 = Ar,\,\,u3 = - 2Az,\,\,\,\,T \to T_{\infty } ,\,\,\,\,C \to C_{\infty } ,\,\,\,\,N \to N_{\infty } ,\,\,as\,\,z \to \infty .\,\,$$

Here, $$\left( {u1\& u3} \right)$$ are components of velocity along *r* and *z* directions respectively, $$p$$ fluid pressure, $$\alpha$$ material parameter, $$g^{*}$$ gravity, $$\beta^{**}$$ volume exception parameter, $$\rho_{m}$$ density microorganism, $$\rho_{p}$$ nanoparticle density of fluid, $$\nu$$ kinematic viscosity, $$D_{B}$$ Brownian motion coefficient, $$\left( {T,C,N} \right)$$ temperature, nanoparticle concentration and swimming microorganisms respectively, $$\left( {T_{\infty } ,C_{\infty } \& N_{\infty } } \right)$$ ambient temperature and ambient nanoparticle concentration and ambient microorganism respectively, $$Kr^{2}$$ rate of chemical reaction coefficient, $$D_{T}$$ thermophoresis diffusion coefficient, $$D_{m}$$ coefficient of swimming microorganisms, $$\sigma^{**}$$ electrical conductivity, $$b$$ chemotaxis constant, $$W_{c}$$ cell swimming speed, $$\gamma$$ material constant parameter, $$E_{a}$$ Arrhenius activation energy and $$\left( m \right)$$ fitted rate constant parameter.

### Similarity transformations

The similarity variables are as follows:

21 $$\begin{aligned} \zeta = & z\sqrt {\frac{A}{\nu }} ,\;\;u1 = Arf^{^{\prime}} \left( \zeta \right),\;\;u3 = - 2\sqrt {A\nu } f\left( \zeta \right),\;\;p = A\mu p^{*} \left( \zeta \right) - \rho \frac{{A^{2} r^{2} }}{2}, \\ \theta \left( \zeta \right) = & \frac{{T - T_{m} }}{{T_{\infty } - T_{m} }},\phi \left( \zeta \right) = \frac{{C - C_{m} }}{{C_{\infty } - C_{m} }},\;\;\chi \left( \zeta \right) = \frac{{N - N_{m} }}{{N_{\infty } - N_{m} }}. \\ \end{aligned}$$

### Dimensionless equations

Non-dimensional equations after applying the transformations are:

22 $$f^{\prime\prime\prime}\left( \zeta \right) - f^{^{\prime}2} \left( \zeta \right) + 2f\left( \zeta \right)f^{\prime\prime}\left( \zeta \right) + M\left( {1 - f^{\prime}\left( \zeta \right)} \right) + 1 + \lambda \left( {\theta - Nr\phi - Nc\chi } \right) = 0,$$

23 $$p^{^{\prime}*} \left( \zeta \right) = - 2f^{\prime\prime}\left( \zeta \right) - 4f\left( \zeta \right)f^{\prime}\left( \zeta \right),$$

24 $$\left( {1 + Rd} \right)\theta^{\prime\prime}\left( \zeta \right) + \Pr \left( {2f\left( \zeta \right)\theta^{\prime}\left( \zeta \right)} \right) + Nb\phi^{\prime}\left( \zeta \right)\theta^{\prime}\left( \zeta \right) + Nt\theta^{^{\prime}2} \left( \zeta \right) = 0,$$

25 $$\phi^{\prime\prime}\left( \zeta \right) + 2Le\Pr f\left( \zeta \right)\phi^{\prime}\left( \zeta \right) + \frac{Nt}{{Nb}}\theta^{\prime\prime}\left( \zeta \right) - Le\Pr \sigma (1 + \delta \theta )^{m} \exp \left( {\frac{ - E}{{1 + \delta \theta }}} \right)\phi = 0,$$

26 $$\chi^{\prime\prime}\left( \zeta \right) + 2Lbf\left( \zeta \right)\chi^{\prime}\left( \zeta \right) - Pe\left[ {\chi^{\prime}\left( \zeta \right)\phi^{\prime}\left( \zeta \right) + \left( {\Omega + \chi \left( \zeta \right)} \right)\phi^{\prime\prime}\left( \zeta \right)} \right] = 0,$$

### Dimension-less boundary conditions

Here the non-dimensional boundary constraints are:

27 $$\begin{aligned} & \Pr f\left( 0 \right) + Me\theta^{\prime}\left( 0 \right),\,f^{\prime\prime}\left( 0 \right) = \lambda^{*} \left\{ {f^{\prime}\left( 0 \right)} \right\}^{n} \left[ {1 - \beta f^{\prime}\left( 0 \right)^{n} } \right]^{{ - \frac{1}{2}}} ,\,\,\,\theta^{\prime}\left( 0 \right) = - Bi\left( {1 - \theta \left( 0 \right)} \right), \\ & \phi^{\prime}\left( 0 \right) + \frac{Nt}{{Nb}}\theta^{\prime}\left( 0 \right) = 0\,\,\chi ,\left( 0 \right) = 1, \\ \end{aligned}$$

28 $$p^{*} \left( 0 \right) = 0,f^{\prime}\left( \infty \right) = 1,\theta \left( \infty \right) = 0,\phi \left( \infty \right) = 0,\chi \left( \infty \right) = 0.$$

### Prominent parameters

The dimensionless parameters are designed bellow:

**Table:** 

Magnetic parameter	$$M = \frac{{\sigma^{**} B_{0}^{2} }}{\sigma a}$$
Prandtl number	$Pr = \frac{{\mu C_{p} }}{\alpha }$
Chemical reaction parameter	$\sigma = \frac{{kr^{2} }}{A}$
Slip parameter	$\lambda^{*} = \frac{k\sqrt \nu }{\mu }\left( {\frac{\pi }{Q}} \right)^{\frac{1}{3}} \frac{{A^{\frac{2}{3}} }}{{A^{\frac{3}{2}} }}$
Radiation parameter	$Rd = \frac{{16T_{\infty }^{3} \sigma^{*} }}{{3k^{*} \alpha }}$
Thermophoresis diffusion	$Nt = \frac{{\tau D_{T} \left( {T_{\infty } - T_{m} } \right)}}{{T_{\infty } \nu }}$
Brownian motion	$Nb = \frac{{\tau D_{B} \left( {\left( {C_{\infty } - C_{m} } \right)} \right)}}{\nu }$
Lewis number	$Le = \frac{\alpha }{{D_{B} }}$
Mixed convection parameter	$$\lambda = \frac{{\beta^{**} g*\left( {1 - C_{\infty } } \right)\left( {T_{\infty } - T_{m} } \right)}}{{aU_{w} }}$$
Generalized slip	$\beta = \beta^{*} k\left( {\frac{\pi }{Q}} \right)^{\frac{1}{3}} rA^{\frac{2}{3}}$
Bouncy ratio parameter	$$Nr = \frac{{\left( {\rho_{p} - \rho_{f} } \right)\left( {C_{\infty } - C_{m} } \right)}}{{\rho_{f} \left( {1 - C_{\infty } } \right)\left( {T_{\infty } - T_{m} } \right)\beta^{**} }}$$
Bioconvection Rayleigh number	$$Nc = \frac{{\gamma *\left( {\rho_{m} - \rho_{f} } \right)\left( {N_{\infty } - N_{m} } \right)}}{{\rho_{f} \left( {1 - C_{\infty } } \right)\left( {T_{\infty } - T_{m} } \right)\beta^{**} }}$$
Activation energy parameter	$E = \frac{{E_{a} }}{{K_{1} T_{\infty } }}$
Bioconvection Lewis number	$Lb = \frac{\nu }{{D_{m} }}$
Peclet number	$Pe = \frac{{bW_{c} }}{{D_{m} }}$
Difference parameter of microorganisms	$\Omega = \frac{{N_{\infty } }}{{N_{\infty } - N_{m} }}$
Biot number	$Bi = \frac{{h_{f} }}{k}\sqrt {\frac{\nu }{A}}$
Melting parameter	$Me = \frac{{c_{p} \left( {T_{\infty } - T_{m} } \right)}}{{L + c_{s} \left( {T_{m} - T_{0} } \right)}}$

## Numerical scheme

The bvp4c solver (shooting method) in MATLAB is utilized to achieve the numerical solution of nonlinear system (22)–(26) with boundary constraints (27) and (28). For this multiple order system of equations are transmuted into first order. The initial approximation with tolerance 10^–6^ is needed for numerical technique. The considered guess approximation must persuade the boundary constraints without disturbing the solution method. The following supposition is helpful to compute the solution:

29 $$\begin{array}{*{20}l} {f = s_{1} ,f_{\zeta } = s_{2} ,f_{\zeta \zeta } = s_{3} ,f_{\zeta \zeta \zeta } = s^{\prime}_{3} ,} \hfill \\ {\theta = s_{4} ,\theta_{\zeta } = s_{5} ,\theta_{\zeta \zeta } = s^{\prime}_{5} ,} \hfill \\ {\phi = s_{6} ,\phi_{\zeta } = s_{7} ,\phi_{\zeta \zeta } = s^{\prime}_{7} ,} \hfill \\ {\chi = s_{8} ,\chi_{\zeta } = s_{9} ,\chi_{\zeta \zeta } = s^{\prime}_{9} ,} \hfill \\ \end{array}$$

30 $$s^{\prime}_{3} \left( \zeta \right) = s_{2}^{2} \left( \zeta \right) - 2s_{1} \left( \zeta \right)s_{3} \left( \zeta \right) - M\left( {1 - s_{2} \left( \zeta \right)} \right) - 1 - \lambda \left( {s_{4} - Nrs_{6} - Ncs_{8} } \right),$$

31 $$p^{^{\prime}*} \left( \zeta \right) = - 2s_{3} \left( \zeta \right) - 4s_{1} \left( \zeta \right)s_{2} \left( \zeta \right),$$

32 $$s^{\prime}_{5} \left( \zeta \right) = \frac{{ - \Pr \left( {2s_{1} \left( \zeta \right)s_{5} \left( \zeta \right)} \right) - Nbs_{7} \left( \zeta \right)s_{5} \left( \zeta \right) - Nts_{5}^{2} \left( \zeta \right)}}{{\left( {1 + Rd} \right)}},$$

33 $$s^{\prime}_{7} \left( \zeta \right) = - 2LePrs_{1} \left( \zeta \right)s_{7} \left( \zeta \right) - \frac{Nt}{{Nb}}s^{\prime}_{5} \left( \zeta \right) + Le\Pr \sigma (1 + \delta s_{4} )^{n} \exp \left( {\frac{ - E}{{1 + \delta s_{4} }}} \right)s_{6} ,$$

34 $$s^{\prime}_{9} \left( \zeta \right) = - 2Lbs_{1} \left( \zeta \right)s_{9} \left( \zeta \right) + Pe\left[ {s_{9} \left( \zeta \right)s_{7} \left( \zeta \right) + \left( {\Omega + s_{8} \left( \zeta \right)} \right)s^{\prime}_{7} \left( \zeta \right)} \right],$$

35 $$\begin{array}{*{20}l} {\Pr s_{1} \left( 0 \right) + Me \cdot s_{5} \left( 0 \right),\,\,s_{3} \left( 0 \right) = \lambda^{*} \left\{ {s_{2} \left( 0 \right)} \right\}^{n} \left[ {1 - \beta s_{2} \left( 0 \right)^{n} } \right]^{{ - \frac{1}{2}}} ,\,\,\,s_{5} \left( 0 \right) = - Bi\left( {1 - s_{4} \left( 0 \right)} \right),} \hfill \\ {s_{7} \left( 0 \right) + \frac{Nt}{{Nb}}s_{7} \left( 0 \right) = 0,\,\,s_{8} \left( 0 \right) = 1,\,\,} \hfill \\ {p^{*} \left( 0 \right) = 0,s_{2} \left( \infty \right) = 1,s_{4} \left( \infty \right) = 0,s_{6} \left( \infty \right) = 0,s_{8} \left( \infty \right) = 0.} \hfill \\ \end{array}$$

### Validation of numerical interpretation

Table 1 is considered to illustrate a comparison with previous studies by^50–52^ under some limitations. Here an excellent concurrence between current results and studies in^50–52^ is obtained.

**Table 1:** Comparative numerical results of $f^{\prime\prime}\left( 0 \right)$ with^50–52^ when $M = 0 = Nr = \lambda = Nc = E = 0 = Pe = Lb = 0 = Bi = 0.$

$\lambda^{*}$	$\beta = 0.0$ ^50^	$\beta = 0.0$ RK-4 method^51^	$\beta = 0.0$ Current results	$\beta = 5.0$ ^50^	$\beta = 5.0$ ^52^	$\beta = 5.0$ Current results
0.01	0.009996	0.009999	0.009999	0.00902	0.009906	0.009905
0.02	0.019927	0.019924	0.019925	0.019561	0.019558	0.019557
0.05	0.049242	0.049246	0.049245	0.047082	0.047088	0.047088
0.10	0.096640	0.096638	0.096638	0.088783	0.088784	0.088784
0.20	0.186041	0.18643	0.18642	0.159723	0.159720	0.159720
0.50	0.414730	0.414732	0.414732	0.311099	0.311098	0.311099
1.0	0.687616	0.687612	0.687615	0.465619	0.465616	0.465618
2.0	0.97046	0.97048	0.97048	0.640374	0.640372	0.640371
5.0	1.211823	1.211820	1.211820	0.872404	0.872403	0.872405
10.0	1.275871	1.275870	1.275871	1.024092	1.0224090	1.0224090

## Physical interpretation

The basic motto of this portion is to illustrate the impacts of involved interesting parameters against velocity field, thermal field, solutal field of species and microorganism profile.

**Figure 2:**
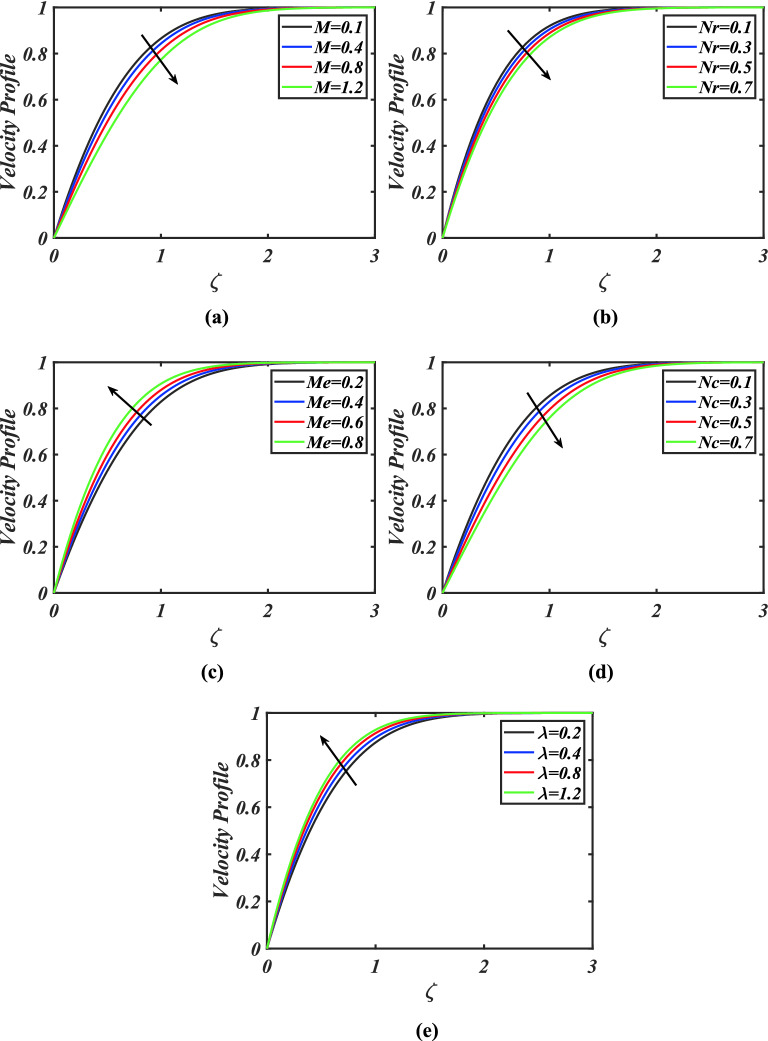
**(a)–(e)** Response of velocity $f^{\prime}$ against varied values of $M,Nr,Me,Nc$ and $\lambda$.

Figure 2a-e are described to ascertain the performance of the velocity field under the impact of magnetic parameter, buoyancy ratio parameter, melting parameter, bioconvection Rayleigh number and mixed convection parameter. The velocity field for buoyancy ratio parameter is pictured in Fig. 2a. A decay in fluid velocity is observed for magnetic parameter. Physically, the larger values of magnetic parameter produce the Lorentz forces which cause a resistance in flow of fluid. The estimations in velocity profile versus buoyancy ratio parameter are explicated in Fig. 2b. It is obvious that, velocity profile is declining function of buoyancy ratio parameter. The trend of melting parameter on nanofluid velocity field is sketched in Fig. 2c. It is clearly observed that flow of fluid improves with intensifying melting parameter. Figure 2d demonstrates the inspirations of velocity field in the presence of slip phenomenon against bioconvection Rayleigh number. It is observed that the velocity of fluid is declined for greater bioconvection Rayleigh number. Physically, for the larger Rayleigh number, bioconvection restricts the up movement of solid particles that emerge in nanofluid for the given buoyancy influence, whereas for the greater buoyancy effect opposes the fluid, resulting in a reduction of fluid motion. Figure 2e expresses the behavior of mixed convection parameter against velocity field. Here we present that velocity field exaggerates for bigger estimations of mixed convection parameter.

**Figure 3:**
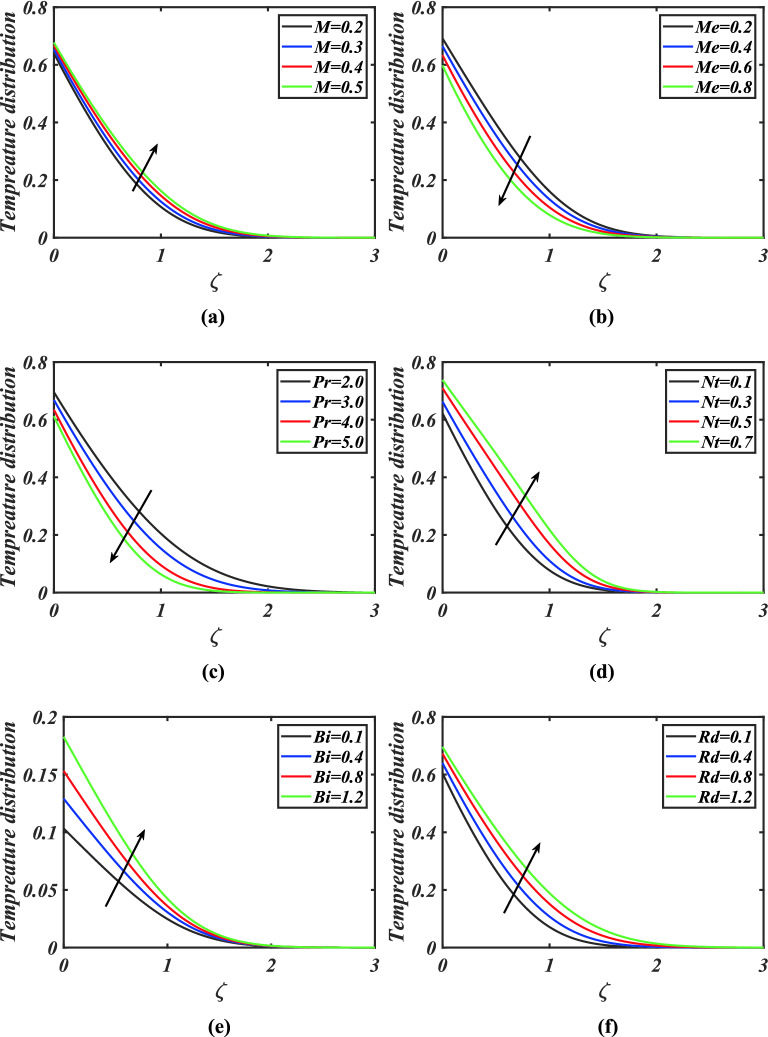
**(a)–(f)** Response of temperature $\theta$ against varied values of $M,Me,Pr,Nt,Bi$ and $Rd$.

Figure 3a-f highlighted the significance of magnetic parameter, melting parameter, Prandtl number, thermophoresis parameter, Biot number and thermal radiation parameter on thermal profile. Figure 3a shows the variation of magnetic parameter on thermal field of nanoparticles. Here the thermal field is boosted by growing estimations of magnetic parameter. The consequence of melting parameter via temperature field is mentioned in Fig. 3b. Here we noted the thermal field diminishes for greater melting parameter. Figure 3c is designed to illustrate the nature of Prandtl number versus temperature field. Physically, the Prandtl number is inversely proportional to thermal diffusivity. Therefore, fluid temperature reduces due to lower thermal diffusivity. Hence thermal field of nanomaterials is declined with growing amount of Prandtl number. Figure 3d is designed to see the effect of thermophoresis parameter on thermal profile. It is concluded that thermal field is improved via larger thermophoresis parameter. The thermophoresis mechanism describes how a temperature gradient causes nanofluid to migrate in a convectively heated surface, resulting in an enhanced heat transfer. Figure 3e reveals that temperature field is boosted by intensifying Biot number. Thermal field is improved with thermal Biot number. Physically, Biot number has a direct relation with heat transfer coefficient. The heat transfer coefficient is increased due to the larger Biot number. Therefore, the temperature distribution is increased. Form Fig. 3f, it can be observed that larger thermal radiation parameter intensifies the temperature field.

**Figure 4:**
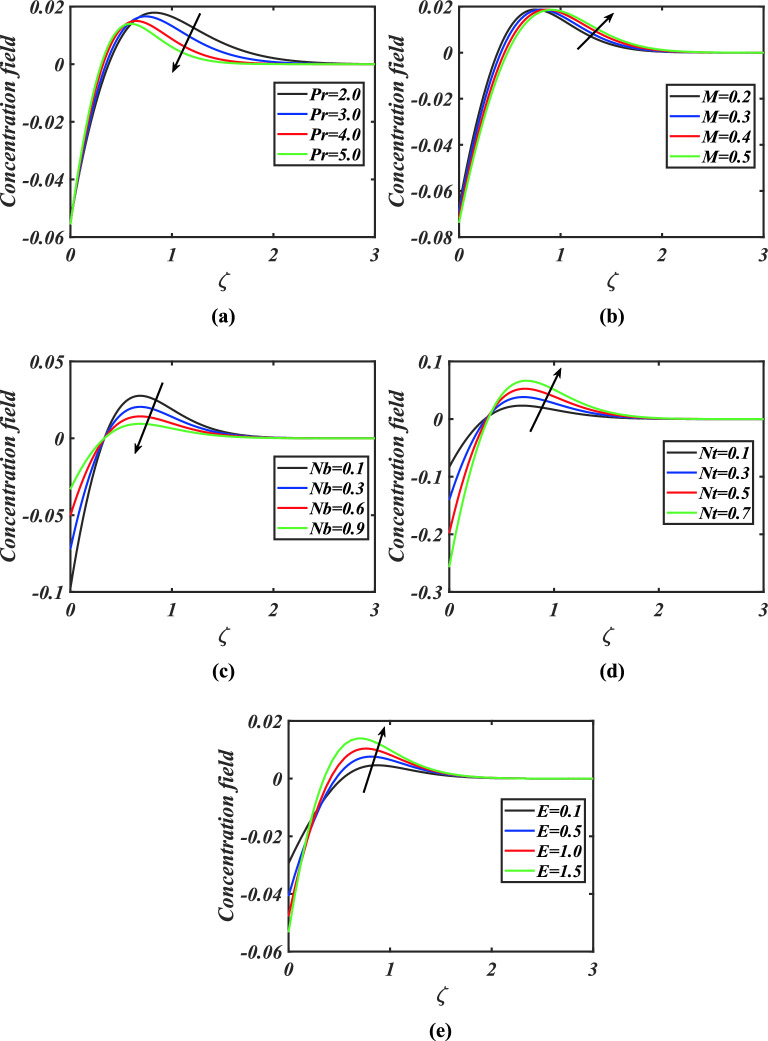
**(a)–(e)** Response of concentration $\phi$ against varied values of $Pr,M,Nb,Nt$ and $E$.

Figure 4a-e are presented to scrutinize the significance of Prandtl number, magnetic parameter, Brownian motion parameter, thermophoresis parameter and activation energy parameter on concentration field of nanoparticles. Figure 4a portrays that concentration diminishes by growing estimations of Prandtl number. Figure 4b visualizes that an augmentation in the magnitude of magnetic parameter causes enhancement in concentration field. Figure 4c demonstrates the impact of Brownian motion parameter against concentration field. Here it can be noticed that greater Brownian motion reduces the concentration of nanoparticles. Figure 4d delineates the behavior of solutal field against thermophoresis parameter. The solutal field is improved via larger thermophoresis parameter. Figure 4e illustrate that the solutal field is increased by escalating amount of activation energy parameter.

**Figure 5:**
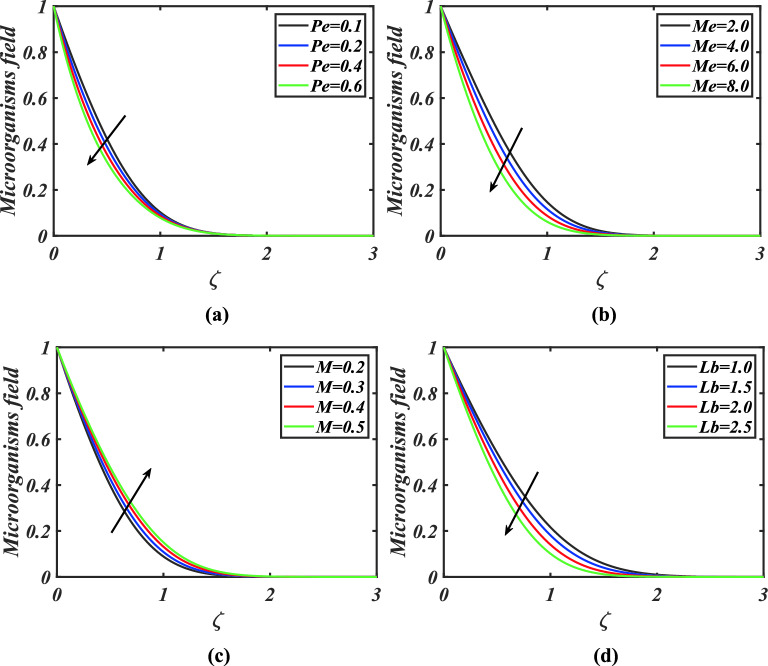
**(a)–(d)** Response of microorganisms $\chi$ against varied values of $Pe,Me,M$ and $Lb$.

Figure 5a-d examine the effects of Peclet number, melting parameter, magnetic parameter and bioconvection Lewis number on microorganism’s field. The microorganism’s field is retarded with improving Peclet number, melting parameter and Lewis number as captured in Fig. 5a, b, d. Physically the bioconvection Lewis number has opposite relation with diffusivity of microorganisms. An increment in bioconvection Lewis number yields weaker diffusivity and so microorganisms’ profile declines. From Fig. 5c we scrutinized that microorganism’s field is an enhancing function of magnetic parameter.

In this portion, the numerical interpretation is discussed in detail. The different variations of involved parameters on skin friction, heat transfer rate, mass transfer rate and microorganism number are mentioned in Tables 2, 3, 4, 5.

**Table 2:** Numerical computations of drag force $- f^{\prime\prime}\left( 0 \right)$ for specific magnitudes of $M$, $Nr$, $\lambda$, $Me$ and $Nc$.

$M$	$Nr$	$\lambda$	$Me$	$Nc$	$- f^{\prime\prime}\left( 0 \right)$
**0.1**	0.1	0.1	0.4	0.1	1.5923
**0.3**					1.6596
**0.5**					1.7241
0.2	**0.2**	0.1	0.4	0.1	1.4991
	**0.4**				1.6265
	**0.6**				1.6375
0.2	0.1	**0.2**	0.4	0.1	1.5207
		**0.3**			1.4991
		**0.4**			1.4774
0.2	0.1	0.1	**0.1**	0.1	1.4307
			**0.2**		1.4020
			**0.3**		1.3758
0.2	0.1	0.1	0.4	**0.2**	1.6162
				**0.4**	1.5958
				**0.6**	1.5753

**Table 3:** Numerical computations of local Nusselt number $- \theta^{\prime}\left( 0 \right)$ for specific magnitudes of $M$, $Nr$, $\lambda$, $Me$, $Nc$, $Pr$, $Bi$, $Nb$, $Nt$ and $Rd$.

$M$	$Nr$	$\lambda$	$Me$	$Nc$	$Pr$	$Bi$	$Nb$	$Nt$	$Rd$	$- \theta^{\prime}\left( 0 \right)$
**0.1**	0.1	0.1	0.4	0.1	2.0	2.0	0.3	0.1	0.5	0.7298
**0.3**										0.7331
**0.5**										0.7362
0.2	**0.2**	0.1	0.4	0.1	2.0	2.0	0.3	0.1	0.5	0.7246
	**0.4**									0.6956
	**0.6**									0.6784
0.2	0.1	**0.2**	0.4	0.1	2.0	2.0	0.3	0.1	0.5	0.7258
		**0.3**								0.7246
		**0.4**								0.7234
0.2	0.1	0.1	**0.1**	0.1	2.0	2.0	0.3	0.1	0.5	0.5766
			**0.2**							0.5821
			**0.3**							0.5931
0.2	0.1	0.1	0.4	**0.2**	2.0	2.0	0.3	0.1	0.5	0.7310
				**0.4**						0.7300
				**0.6**						0.7290
0.2	0.1	0.1	0.4	0.1	**3.0**	2.0	0.3	0.1	0.5	0.7315
					**4.0**					0.8453
					**5.0**					0.8838
0.2	0.1	0.1	0.4	0.1	2.0	**3.0**	0.3	0.1	0.5	0.8620
						**4.0**				0.9516
						**5.0**				1.0177
0.2	0.1	0.1	0.4	0.1	2.0	2.0	**0.2**	0.1	0.5	0.7077
							**0.4**			0.6927
							**0.6**			0.6716
0.2	0.1	0.1	0.4	0.1	2.0	2.0	0.3	**0.2**	0.5	0.6958
								**0.4**		0.6720
								**0.6**		0.6483
0.2	0.1	0.1	0.4	0.1	2.0	2.0	0.3	0.1	**0.1**	0.7974
									**0.3**	0.7474
									**0.6**	0.6908

**Table 4:** Numerical computations of local Sherwood number $\phi^{\prime}\left( 0 \right)$ for specific magnitudes of $M$, $Nr$, $\lambda$, $Me$, $Nc$, $Pr$, $E$, $Nb$ and $Nt$.

$M$	$Nr$	$\lambda$	$Me$	$Nc$	$Pr$	$E$	$Nb$	$Nt$	$\phi^{\prime}\left( 0 \right)$
**0.1** **0.3** **0.5**	0.1	0.1	0.4	0.1	2.0	0.1	0.3	0.1	0.2433 0.2444 0.2454
0.2	**0.2** **0.4** **0.6**	0.1	0.4	0.1	2.0	0.1	0.3	0.1	0.2415 0.2318 0.2212
0.2	0.1	**0.2** **0.3** **0.4**	0.4	0.1	2.0	0.1	0.3	0.1	0.2419 0.2415 0.2411
0.2	0.1	0.1	**0.1** **0.2** **0.3**	0.1	2.0	0.1	0.3	0.1	0.1922 0.2034 0.2114
0.2	0.1	0.1	0.2	**0.2** **0.4** **0.6**	2.0	0.1	0.3	0.1	0.2437 0.2433 0.2430
0.2	0.1	0.1	0.2	0.1	**3.0** **4.0** **5.0**	0.1	0.3	0.1	0.2438 0.2818 0.2946
0.2	0.1	0.1	0.2	0.1	2.0	**0.2** **0.4** **0.6**	0.3	0.1	0.2359 0.2041 0.1876
0.2	0.1	0.1	0.2	0.1	2.0	0.1	**0.2** **0.4** **0.6**	0.1	0.3539 0.1769 0.1180
0.2	0.1	0.1	0.2	0.1	2.0	0.1	0.3	**0.2** **0.4** **0.6**	0.4639 0.4422 0.4045

**Table 5:** Numerical estimations of local microorganisms density number $- \chi^{\prime}\left( 0 \right)$ for specific magnitudes of $M$, $Nr$, $\lambda$, $Me$, $Nc$, $Pe$ and $Lb$.

$M$	$Nr$	$\lambda$	$Me$	$Nc$	$Pe$	$Lb$	$- \chi^{\prime}\left( 0 \right)$
**0.1** **0.3** **0.5**	0.1	0.1	0.4	0.1	0.1	3.0	1.4167 1.4274 1.4373
0.2	**0.2** **0.4** **0.6**	0.1	0.4	0.1	0.1	3.0	1.4003 1.3932 1.3819
0.2	0.1	**0.2** **0.3** **0.4**	0.4	0.1	0.1	3.0	1.4040 1.4003 1.3965
0.2	0.1	0.1	**0.1** **0.2** **0.3**	0.1	0.1	3.0	1.0806 1.0991 1.1013
0.2	0.1	0.1	0.4	**0.2** **0.4** **0.6**	0.1	3.0	1.4206 1.4174 1.4142
0.2	0.1	0.1	0.4	0.1	**0.2** **0.3** **0.4**	3.0	1.7828 1.8041 1.8254
0.2	0.1	0.1	0.4	0.1	0.1	**1.0** **1.5** **2.0**	0.9896 1.2116 1.4086

**Figure 6:**
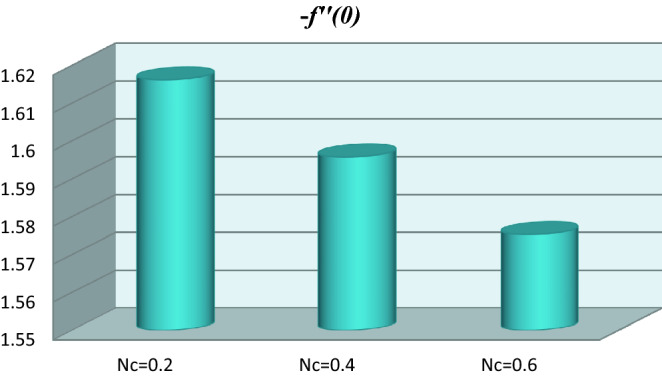
Local skin friction coefficient versus bioconvection Rayleigh number.

The graphical representation of local skin friction coefficient for dissimilar variations of bioconvection Rayleigh number is shown in Fig. 6. Here we observed the local skin friction coefficient reduces for greater bioconvection Rayleigh number.

**Figure 7:**
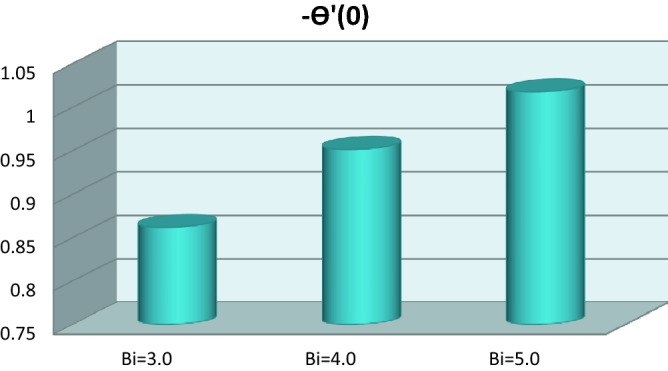
Local Nusselt number versus Biot number.

The graphical behavior of local Nusselt number is depicted in Fig. 7. The local Nusselt number is boosted with growing Biot number.

**Figure 8:**
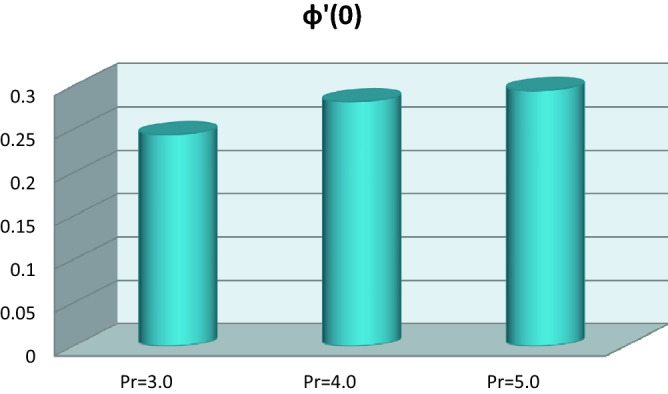
Local Sherwood number versus Prandtl number.

The graphical interpretation of local Sherwood number with Prandtl number is given in Fig. 8. The Prandtl impact boosts the local Sherwood number.

**Figure 9:**
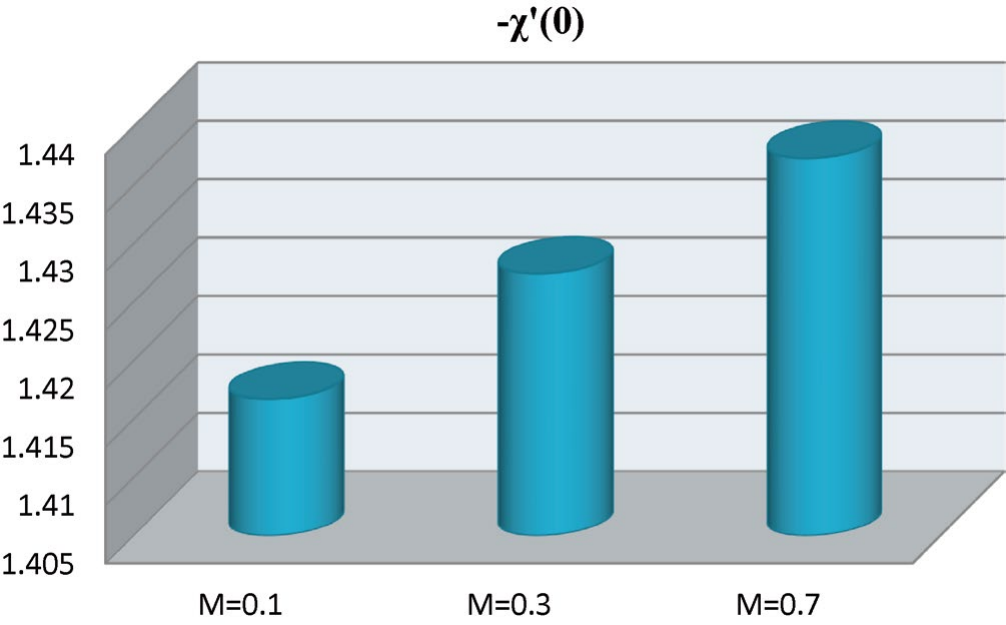
Local microorganism’s density number versus magnetic parameter.

The graphical behavior of magnetic parameter against local microorganism’s density number is shown in Fig. 9. Here the local density number increases via larger estimations of magnetic parameter.

## Conclusions

A novel analysis is reported for bioconvective nanofluid flow through lubricated surface with melting phenomenon. The significance of thermal radiation and activation energy under generalized slip impact has been scrutinized. The problem is structured by partial differential equations with appropriate boundary restrictions and then transmute to ODEs by applying similarity transformations. The dimensionless system of ODEs then resolved numerically by applying bvp4c solver via Lobatto-IIIa formula. The main concluding remarks of the current analysis are summarized below:

- The validation of the current result of skin friction with previous research^50–52^ is conducted in Table 1.
- The velocity field is enhanced due to the mounted mixed convection parameter.
- The velocity profile is improved due to enhanced melting parameter.
- The velocity profile is reduced for enhancing bioconvection Rayleigh number and magnetic parameter impacts.
- The temperature distribution is escalated due to the improved thermophoresis parameter.
- The temperature field is enhanced by the growing Biot number while declines for greater values of Prandtl number.
- The volumetric concentration of nanoparticles is increased due to the increased activation energy.
- The concentration profile is enhanced due to larger variations of magnetic parameter.
- The microorganism field is diminished via larger Peclet number.
- The microorganisms profile is reduced due to increment in bioconvection Lewis number.
- Greater melting parameter declines the density number of microorganism’s profile.
- The bioconvective nanofluid flow over lubricated disk is more suitable for heat transfer enhancement. The current results are more useful for thermodynamics and heat transfer problems.

## Abbreviations

(*u*1&*u*3): Velocity components
*p*: Pressure
(*r*,*z*): Coordinates system
*g*^*^: Gravity
*β*^**^: Volume exception
*ρ*_*m*_: Microorganisms’ density
*ρ*_*p*_: Nanoparticle’s density
*ν*: Kinematic viscosity
*D*_*T*_: Thermophoresis diffusion coefficient
*σ*: Electrical conductivity
*W*_*c*_: Cell swimming speed
*E*_*a*_: Activation energy coefficient
*n*: Power-law index
*k*^*^: Mean absorption coefficient
*M*: Magnetic parameter
*Pr*: Prandtl number
*σ*: Chemical reaction parameter
*λ*^*^: Slip parameter
*Rd*: Radiation parameter
*Nt*: Thermophoresis diffusion
*Nb*: Brownian motion
*Bi*: Biot number
Ω: Microorganisms difference parameter
*D*_*B*_: Brownian motion coefficient
*T*: Temperature
*C*: Concentration
*N*: Microorganisms
$$T_{\infty }$$: Ambient temperature
$$C_{\infty }$$: Ambient concentration
$$N_{\infty }$$: Ambient microorganisms
*Kr*^2^: Chemical reaction coefficient
*D*_*m*_: Microorganisms’ coefficient
*b*: Chemotaxis constant
*γ*: Material constant
*m*: Fitted constant rate
*σ*^*^: Stefan Boltzmann constant
*Le*: Lewis number
*λ*: Mixed convection parameter
*β*: Generalized slip
*Nr*: Bouncy ratio parameter
*Nc*: Bioconvection Rayleigh number
*E*: Activation energy parameter
*Lb*: Bioconvection Lewis number
*Pe*: Peclet number
*Me*: Melting parameter
